# Strategies to reduce CMV infectivity in breastmilk to preterm babies – impact on transmission, nutrients, and bioactivity: a systematic review and meta-analysis

**DOI:** 10.1038/s41372-025-02254-9

**Published:** 2025-03-27

**Authors:** Akshita Singh, Adam Bartlett, Vanessa Clifford, Brendan McMullan, Pamela Palasanthiran

**Affiliations:** 1 University of New South Wales, Sydney, NSW Australia; 2 Department of Infectious Diseases, Sydney Children’s Hospitals Network, Randwick, NSW Australia; 3 Department of Paediatrics, University of Melbourne, Parkville, VIC Australia; 4 Infection and Immunity, Murdoch Children’s Research Institute, Parkville, VIC Australia; 5 Laboratory Services, Royal Children’s Hospital, Parkville, VIC Australia; 6 Australian Red Cross Lifeblood, West Melbourne, VIC Australia

**Keywords:** Viral infection, Infection, Viral infection, Paediatrics

## Abstract

**Introduction:**

Postnatal CMV infection (pCMV) acquired via breastmilk is associated with morbidity and mortality in vulnerable infants (<32 weeks or <1500 g). ‘Treatment’ of breast milk reduces CMV infectivity but quantitative impact on transmission, viral loads, bioactive and nutritional elements is unknown. We conducted a systematic review and meta-analysis to assess how each method impacts CMV transmission rates and viral loads and provide a narrative review of their impact on nutritional and bioactive elements.

**Methods:**

Three search strategies for MEDLINE and EMBASE were used to identify articles studying the impact of treatment methods on CMV transmission (Arm A), nutritional elements (Arm B) and bioactive elements (Arm C). Two authors independently screened articles against inclusion and exclusion criteria. Included articles underwent quality assessment using the ROBINS-I tool. Quantitative analysis of data extracted from arm A is presented, alongside narrative reviews of arms B and C.

**Results:**

Twenty-six studies (*n* = 3024 infants) were included for arm A. Heat treatment methods and freeze thawing resulted in 82% and 53% reduction CMV transmission respectively, compared to untreated milk. Correlation between viral load magnitude and transmission risk was not significant. Macronutrients remained largely stable after treatment, but bioactive elements were significantly degraded by heat treatment methods. High Pressure Processing was significantly better at preserving bioactive elements compared to heat treatment.

**Conclusion:**

Heat treatment is most effective in reducing CMV infectivity in breastmilk but is associated with higher degradation of bioactive elements, whilst microwave irradiation and HPP eliminate CMV in breastmilk and preserve its immunological integrity.

## Introduction

Postnatal cytomegalovirus infection (pCMV) in newborns, predominantly acquired via breastmilk feeding [1] is common, occurring at rates ranging from 5.7 to 58.6% [1–3]. pCMV in vulnerable pre-term newborns, i.e. <32 weeks gestational age and/or <1500 g birthweight [1, 4] may result in significant complications including haematological disease, gastrointestinal dysfunction, necrotising enterocolitis, pneumonia or a CMV-Sepsis Like Syndrome (CMV-SLS), including death [5]. pCMV increases length of hospital stay and the incidence of bronchopulmonary dysplasia, which in-turn results in a five-fold increase in mortality [6].

Thus preventing, or minimising CMV acquisition in vulnerable babies is relevant and significant. As premature babies are dependent on ‘mother’s-own-milk’ for nutrition and immunological benefits, exclusion of breast milk feeding is undesirable [7]. A primary prevention strategy is the use of various ‘treatment methods’ to reduce CMV bioburden in breastmilk to reduce transmission risks. Available treatment methods include Freeze Thawing (−20 °C for 3–5 days), Pasteurization methods and Irradiation. Pasteurization methods include Low Temperature Long Time pasteurisation, also known as Holder Pasteurisation (HoP, 62.5 °C for 30 min) [4] and High Temperature Short Time (HTST, 72 °C for 5–15 s) [8]. Novel treatments include irradiation via ultraviolet-C or microwaves at variable powers and durations. High Pressure Processing is a well-established treatment method in the food industry, recently explored to eradicate CMV in breastmilk [9, 10].

However, these treatment methods also deplete breastmilk’s naturally occurring nutritive (lipids, lactose) and protective constituents (immunoglobulins, enzymes). Ninety percent of the infant’s energy supply is sourced from lactose, triglycerides, and other complex lipids [8]. Lactoferrin plays a role in intestinal growth and contributes to anti-microbial properties of milk along with lysozyme through immunomodulation [11]. Maternal antibodies are essential for antigen specific immune reactions [11, 12] and is key in protecting infants from acquiring pCMV. Bile salt stimulated lipase (BSSL) is essential for digestion of breastmilk lipids, to compensate for the inadequately developed digestive ability [12]. It enhances metabolism and absorption of vitamins, which also contributes to overall development of the infant. Absence of this dynamic supply from ‘treated’ milk results in increased susceptibility to metabolic syndrome, asthma, and other developmental difficulties [13]. This study therefore focused on the impact of treatment methods on selected elements: lactose, lipids, lactoferrin, lysozyme, sIgAs, and BSSL.

Whist existing reviews have explored the effectiveness of processing breastmilk in reducing CMV transmission, the quantitative impact of the treatment methods on virological correlates of CMV, nutritional and immunological elements in breastmilk remains unknown. Quantification of the impact of interventions would provide perspective on the magnitude of CMV transmission reduction in addition to the degree of loss of nutrition and protection.

We conducted a systematic review and meta-analysis to study:

1. The quantitative impact of treatment methods on CMV transmission rates via breast milk
2. The quantitative impact of treatment methods on CMV viral loads in breast milk
3. The impact of treatment methods on selected nutritional and immunological components of breastmilk.

## Methodology

### Systematic review on outcomes related to CMV reduction and transmission rates

**Fig. 1:**
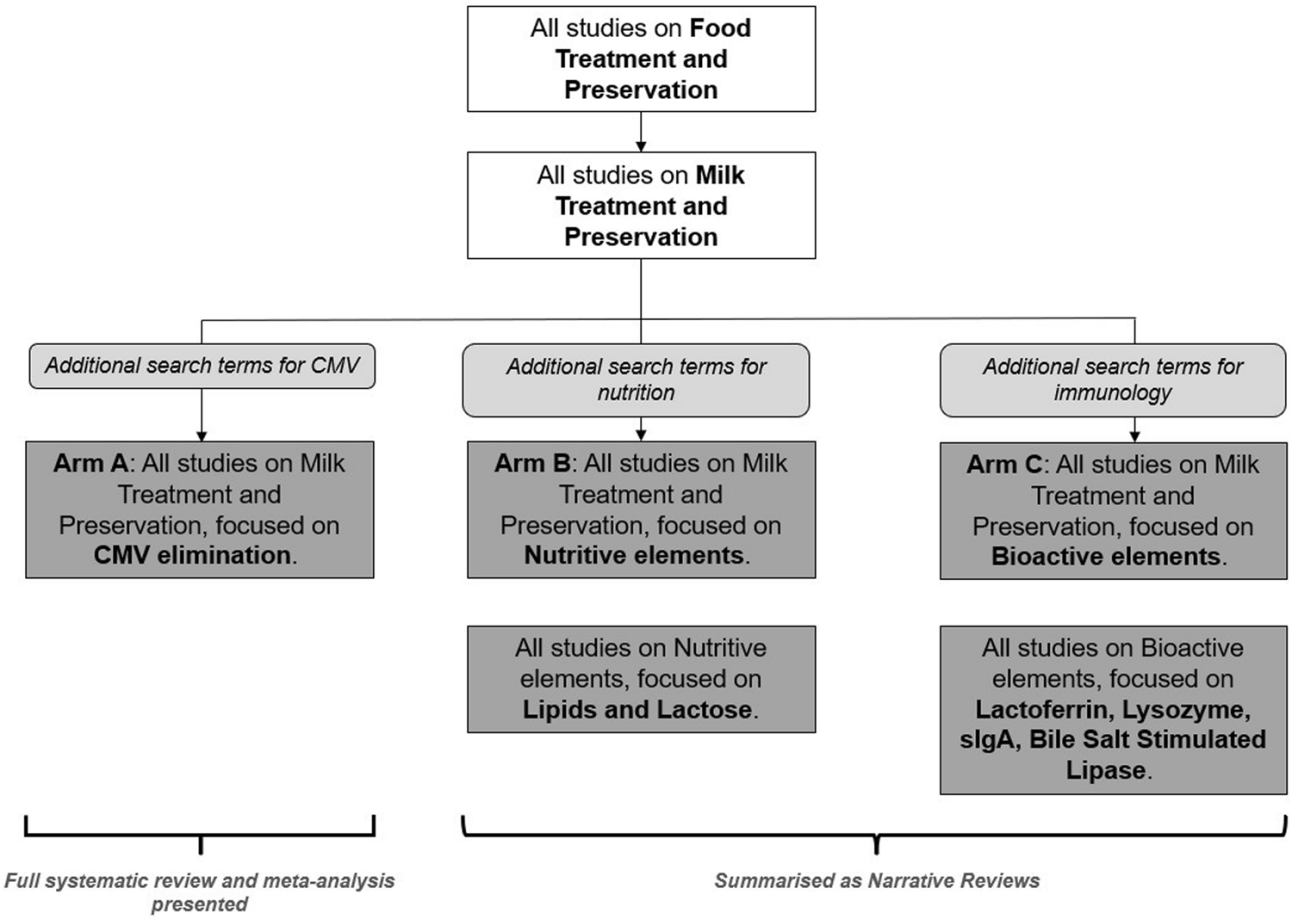
Search strategy for systematic review of articles focused on breastmilk treatments methods to eliminate CMV. The structure was used for searches in both Medline and EMBASE. Additional search terms to narrow the search were added at each level. CMV Cytomegalovirus, sIgA secretory Immunoglobulin A.

This study had three arms (Fig. 1). Arm A was designed to identify articles that studied impact of breastmilk treatment on virological correlates of CMV transmission (viral burden) and on pCMV incidence rates. Arms B and C identified research that study the impact of breastmilk treatment on relevant nutritional and bioactive elements. A literature search was conducted on MEDLINE and EMBASE using a search strategy (Supplementary Material 1). A systematic review and data analyses of Arm A were presented. Arm B and C were summarised as narrative reviews. Prominent search terms included “Cytomegalovirus”, “Postnatal Cytomegalovirus”, “Milk, Human”, “Infant, Premature”, “Pasteurization”, “Irradiation” and “Freezing”. A single article was included from a personal library as it was not accessible in the databases included in this study [9]. The study was registered on the International Prospective Register of Systematic Reviews (PROSPERO) (Registration ID: CRD42022268371).

For each arm, duplicates were removed using Endnote and imported into Rayyan [14] for title and abstract screening. Two authors independently screened the articles against inclusion and exclusion criteria (A.S. and P.P for arm A, A.S and A.B for arms B and C).

The selection criteria for Arm A included articles that were (i) focused on vulnerable infants (<32 weeks gestational age and/or <1500 g birthweight) and their mothers, (ii) reported pCMV infection incidence, breastmilk transmission of CMV and (iii) focused thermal and non-thermal methods of treatment. We excluded articles focused on (i) term infants, (ii) congenital CMV infections, (iii) other modes of transmission, (iv) clinical treatment and management of CMV. We also excluded case reports, reviews, conference abstracts and opinion articles. Thirty-six articles were included for full text reviewing. The articles were assessed for quality and risk of bias, using the ROBINS-I tool (2016) [15]. Articles were assessed for bias across seven domains and an overall risk category was assigned from one of the following: (i) Low: the study is comparable to a randomized trial, (ii) Moderate: The study provides good evidence but not comparable to a randomized trial, and (iii) Serious: The study had some important problems impeding results [15].

**Fig. 2:**
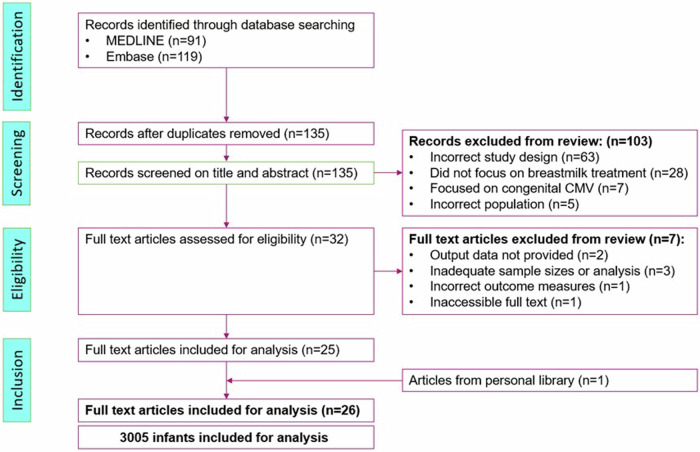
PRISMA flowchart for systematic review in arm A. Articles filtered from search strategies underwent title and abstract screening by two reviewers independently. Conflicted articles were included or excluded on a case-by-case discussion.

Articles in non-English languages with inaccessible full texts were excluded from the study, as a risk of bias assessment could not be performed. Seven articles were excluded after a full text review. Twenty-six articles were included in the analysis. The review process is shown in the PRISMA flowchart (Fig. 2).

### Arm A - meta-analyses

Studies on clinical outcomes of CMV transmission were grouped into studies that used 1) untreated (fresh) breastmilk, 2) frozen breast milk, 3) pasteurized breast milk 4) combined formulations to feed their infants. Proportional meta-analysis was run on each subgroup to obtain pooled effect sizes (Supplementary Material 2) and compared visually. An I^2^ statistic was calculated to assess heterogeneity. The meta-analysis was completed on the Joanna Briggs Institute (JBI) SUMARI online software tool for Systematic Reviews. The Freeman turkey transformation was the statistical method applied along with a random effects model [16].

An ‘infected’ milk sample was defined by a sample that returned a positive CMV PCR, viral culture, or cytopathic effect demonstrating presence of CMV. Some laboratory experiments documented presence of CMV DNA quantitatively (copies/mL) by PCR [4, 17, 18]. Two studies cultured milk samples for cytopathic effect of CMV [19, 20]. Others attempted to isolate CMV from the cultures and provided counts of Plaque Forming Units (PFU) per mL [21], Tissue Culture Infective Dose (TCID50) per mL [22], or their logarithmic forms [18].

As studies used a variety of methods to ascertain the amount of CMV present in samples, a proportional change in amount of CMV in samples of each study effect was calculated. If a sample returned negative cultures or PCR assays, CMV was considered eradicated.

Change in amount of CMV was calculated manually using the formula:

$${Change\; in\; amount} = \frac{{final\; viral\; load},{PFUs\; per\; ml},{or\; cytopathic\; effect}-{initial\; viral\; load},{PFUs\; per\; ml},{or\; cytopathic\; effect}}{{initial\; viral\; load},{PFUs\; per\; ml},{or\; cytopathic\; effect}} \times 100\,$$

Where possible, the conversion factor: 1 TCID50/mL = 0.7 PFU/mL [23] was used to streamline quantitative data. A summary of treatment methods on amount of CMV in breastmilk was tabulated.

A CMV seropositive mother was considered a ‘transmitting’ mother if provision of her CMV infected breastmilk led to an identifiable pCMV infection. To investigate the correlation of magnitude of viral loads and pCMV infection, studies reporting mean viral loads from successful transmitting mothers and non-transmitting mothers were identified. Mean viral loads and their respective standard deviations were used in a fixed effects comparative meta-analysis model in SPSS Statistics for Windows, version 28. Statistical level of significance was *p* ≤ 0.05. In-depth statistical manipulation to standardize outcome measures, units and standard errors have been detailed in Supplementary Material 4.

## Results

### Arm A: systematic review

One hundred and thirty-five articles were identified for title and abstract screening and 26 articles were included for data extraction and analysis (Table 1). Fifteen were cohort studies investigating clinical outcomes while eleven were interventional laboratory experiments. Articles were screened for bias in seven risk domains (Table 2). Six articles were deemed at high risk of bias in one or more domains [19, 20, 24–26], 11 were deemed moderate [4, 18, 27–35], and 10 deemed low risk of bias [9, 17, 21, 22, 36–41].

**Table 1:** Summary of characteristics of articles included for analysis in systematic review of arm A.

S.No	Author	Year	Study design	Treatment methods explored	Location	Population	N	BW	GA	PP
1	Welsh et al. [18]	1979	Interventional Lab Experiment	Pasteurization: (62.5 °C for 30 min), Freeze-thawing: (−15 °C for 10 days)	Australia	Mothers	NR	NA	NA	1 to 28 d pp
2	Dworsky et at. [19]	1982	Interventional Lab Experiment	Pasteurization: (62 °C for 30 min), Freeze-thawing: (−20 °C for up to 7 days)	US	Mothers	32	NA	NA	2 - 18 wks pp
3	Hamprecht et al. [4]	2004	Interventional Lab Experiment	Pasteurization: (62.5 °C for 30 min), Freeze-thawing: (−20 °C for 18 h, 4 days and 10 days)	Germany	Mothers (preterm and term)	6	NA	NA	NR
4	Yoo et al. [28]	2015	Retrospective Cohort Study	Pasteurization: 62 °C for 30 min, Freeze-thawing: (−20 °C for 3 days)	Korea	Infants	385	<1000	NR	NA
5	Stock et al. [29]	2015	Retrospective Cohort Study	Pasteurization: (62.5 °C for 30 min)	Austria	Infants	323	NR	<32	NA
6	Donalisio et al. [30]	2018	Interventional Lab Experiment	Pasteurization: (62.5 °C for 30 min)	Italy	Mothers	18	NR	NR	1 – 15 d pp
7	Gaya et al. [32]	2021	Interventional Lab Experiment	Pasteurization: (63 °C for 1, 2, 10, 30 min)	Italy	Donor	NR	NA	NA	NA
8	Bapistella et al. [31]	2019	Prospective Cohort Study	Pasteurization: (62 °C for 5 s)	Germany	Infants and Mothers (seropositive)	87	<1500	<32	NA
9	Buxmann et al. [35]	2009	Prospective Longitudinal Cohort Study	Freeze-thawing (−18 °C for various durations)	Germany	Infants	58	NA	<31	NA
10	Wakabayashi et al. [42]	2012	Prospective Cohort Study	Freeze-thawing (−20 °C for various durations)	Japan	Infants	11	<1500	NR	NA
11	Omarsdottir et al. [24]	2015	Prospective Longitudinal Cohort Study	Freeze-thawing (−20 °C for 3 days)	Sweden	Infants	140	NR	<28	NA
12	Hosseini et al. [16]	2016	Interventional Lab Experiment	Freeze-thawing (−20 °C for 3 days)	Iran	Infants and Mothers	25	<2000	<32	NA
13	Balcells et al. [25]	2016	Prospective Observational study	Freeze-thawing (−20 °C for 3 days)	Spain	Infants	981	<1500	<32	NR
14	Sam et al. [17]	2018	Prospective Cohort Study	Freeze-thawing (−20 °C for 4 days up to 90 days)	US	Mothers	6	NR	NR	NR
15	Lloyd et al. [21]	2016	Interventional Lab Experiment	UV-C Irradiation (254 nm for 10 s at 1 to 5 cm)	Australia	Donor	NR	NR	NR	NR
16	Ben-Shoshan et al. [39]	2016	Interventional Lab Experiment	Microwave Irradiation (500 W and 750 W for 10 s)	Israel	Mothers	31	NR	NR	NR
17	Mikawa et al. [20]	2019	Interventional Lab Experiment	Microwave Irradiation (500 W for 20, 30, 40, 60 s)	Japan	None (created milk from formula)	NR	NA	NA	NA
18	Maschmann et al. [40]	2019	Interventional Lab Experiment	High Temperature Short Time (72 °C for 5 s)	Germany	Mothers	41	NR	NR	NA
19	Pitino et al. [9]	2022	Interventional Lab Experiment	High Pressure Processing ((350Mpa, 500 Mpa and 600 Mpa for 8 and 10 min	Canada	Donor	1	NR	NR	NR
20	Hernandez-Alverado et al. [33]	2021	Prospective blinded Surveillance Study	Untreated	US	Infants	200	<1500	NR	NA
21	Jim et al. [36]	2004	Prospective Observational study	Untreated	Taiwan	Infants	42	<1500	<35	NA
22	Jim et al. [26]	2009	Prospective Observational Study	Untreated	Taiwan	Infants	23	<1500	<35	NA
23	Hayashi et al. [23]	2011	Prospective Longitudinal Cohort Study	Untreated	Japan	Infants	27	<1000	<28	NA
24	Josephson et al. [27]	2014	Prospective Cohort Study	Untreated	Georgia	Infants	536	<1700	NR	NA
25	Romero Gomez et al. [38]	2015	Prospective Longitudinal Cohort Study	Untreated	Spain	Infants	160	NR	<32	NA
26	Volder et al. [34]	2021	Prospective Cohort Study	Untreated	Denmark	Infants	26	NR	<32	NA

*N* Number of infants that were recruited by the study, *BW* Birthweight criteria used by the study to recruit infants, *GA* Gestational age criteria used by the study to recruit infants, *PP* Time post-partum – Time after delivery at which point milk samples were collected from the mother, *NA* Not Applicable, *NR* Not reported.

**Table 2:** Risk of bias assessment of articles included for analysis in systematic review of arm A.

		Risk domains							
	Author	Risk of Bias due to confounding	Risk of Bias in selection of participants into study	Risk of Bias in classification of interventions	Risk of Bias due to deviation from intended interventions	Risk of Bias due to missing data	Risk of Bias in measurement of outcomes	Risk of Bias in selection of the reported result	Overall
1	Welsh et al. [18]	Serious	Low	No information	Low	Moderate	Moderate	Moderate	Serious
2	Dworsky et al. [19]	Serious	Moderate	Moderate	Moderate	Low	Moderate	Moderate	Serious
3	Hamprecht et al. [4]	Low	Low	Low	Low	Moderate	Low	Moderate	Moderate
12	Yoo et al. [28]	Moderate	Low	Low	Low	Low	Low	Low	Moderate
13	Stock et al. [29]	Low	Low	Low	Low	Moderate	Low	Low	Moderate
18	Donalisio et al. [30]	Low	Low	Low	Moderate	Low	Low	Low	Moderate
23	Gaya et al. [32]	Moderate	Low	Low	Low	Low	Low	Low	Moderate
20	Bapistella et al. [31]	Low	Low	Low	Moderate	Moderate	Low	Low	Moderate
5	Buxmann et al. [35]	Low	Low	Low	Low	Low	Low	Low	Low
8	Wakabayashi et al. [42]	Low	Low	Low	Low	Low	Low	Low	Low
11	Omarsdottir et al. [24]	Moderate	Low	Low	Serious	Serious	Moderate	Serious	Serious
14	Hosseini et al. [16]	Low	Low	Low	Low	Low	Low	Low	Low
16	Balcells et al. [25]	Low	Low	Serious	Low	Low	Low	Low	Serious
19	Sam et al. [17]	Low	Low	Low	Low	Moderate	Low	Low	Moderate
15	Lloyd et al. [21]	Low	Low	Low	Low	Low	Low	Low	Low
17	Ben-Shoshan et al. [39]	Low	Low	Low	Low	Low	Low	Low	Low
22	Mikawa et al. [20]	Low	Low	Low	Low	Low	Low	Low	Low
21	Maschmann et al. [40]	Low	Low	Low	Low	Low	Low	Low	Low
24	Hernandez-Alverado et al. [33]	Moderate	Low	Low	Low	Moderate	Low	Low	Moderate
6	Jim et al. [36]	Low	Low	Low	Low	Low	Low	Low	Low
4	Jim et al. [26]	Low	Low	Low	Low	Low	Low	Moderate	Moderate
7	Hayashi et al. [23]	Low	Low	Low	Serious	Low	Low	Low	Serious
9	Josephson et al. [27]	Moderate	Low	Low	Moderate	Moderate	Low	Low	Moderate
10	Romero Gomez et al. [38]	Low	Low	Low	Low	Low	Low	Low	Low
25	Volder et al. [34]	Low	Low	Low	Moderate	Low	Low	Low	Moderate
26	Pitino et al. [9]	Low	Low	Low	Low	Low	Low	Low	Low

Interpretation as provided in the ROBINS-I detailed guide:

Low R.O.B: The study is comparable to a well-performed randomized trial.

Moderate R.O.B: The study provides sound evidence for a non-randomized study but cannot be considered comparable to a randomized trial.

Serious R.O.B: The study has some important problems.

In accordance, clinical study participants included vulnerable infants (gestational age <32 weeks, birthweight <1500 g), mothers of vulnerable infants, or mother infant pairs. Amongst laboratory studies, 3 investigated de-identified donor milk samples from milk banks [9, 22, 33], 1 used formula to create milk samples [21], while the rest recruited mothers or mother infant pairs to obtain breastmilk at different time-points [19, 20]. Cumulatively, 251 mothers and 3024 vulnerable infants were included in our quantitative analysis.

### Meta-analyses

**Fig. 3:**
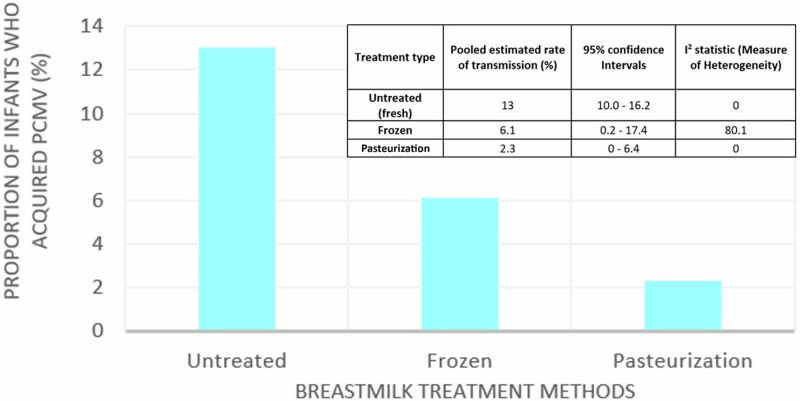
Pooled rates of transmission of CMV. Y-axis represents the proportion of infants who were identified to have pCMV via a positive blood/urine/saliva PCR amongst all infants exposed to CMV infected breastmilk when the breastmilk was untreated (fresh), frozen, or pasteurised. Numerical values used to generate graph are shown on the top right.

Clinical studies on pCMV transmission explored freezing and pasteurisation of breast milk as the ‘treatment’ methods [24–30, 34, 35, 37, 39, 42, 43]. Proportional meta-analysis of the three subgroups showed that CMV transmission rates for untreated breast milk, freeze-thawing and pasteurisation methods (including HoP and short-term pasteurization) was 13%, 6.1% and 2.3% respectively (Fig. 3).

Data on the reduction of viral burden in breast milk by each method is detailed in Table 3. Notable observations include the high efficacy (100%) of HoP, HTST and microwave irradiation in eradicating CMV [4, 21, 40, 41].

**Table 3:** Effect of treatment methods on CMV infectivity of breastmilk.

Name of treatment method	Relevant articles	Results	Inference
Freezing	Welsh et al. [18], Dworsky et al. [19], Hamprecht et al. [4], Hosseini et al. [16], Sam et al. [17]	Mean viral infectivity was reduced by 90% in some studies but increased by 10% in others	Evidence regarding efficacy of freezing is conflicting. Freezing is ineffective in reducing viral loads to non-transmissible levels.
Holder Pasteurization (HoP)	Gaya et al. [32], Hamprecht et al. [4], Dworsky et al. [19]	Viral Infectivity reduction = 100% in all studies	Low Temperature Long Time conditions of HoP are most effective in eradicating CMV.
High Temperature Short Time (HTST)	Maschmann et al. [40]	Mean viral infectivity was reduced by 100% at 72 degrees for 5 s.	HTST is effective at eradicating CMV. More evidence is needed to corroborate.
Microwave Irradiation	Mikawa et al. [20], Ben-Shoshan et al. [39]	Mean Viral Infectivity reduced by about 90% at 500 W, 30 s, and by 100% at 750 W, 30 s.	Microwave Irradiation is effective at eradicating CMV at high powers. More evidence is needed to corroborate.
UV-C Irradiation	Lloyd et al. [21]	Mean viral infectivity was reduced by 54.5%	UV-C irradiation can significantly reduce CMV but lacks efficacy to reduce it to non-transmissible levels. More evidence is needed to corroborate.
High Pressure Processing	Pitino et al. [9]	Mean viral infectivity was reduced to undetectable levels (manually estimated to >89% reduction in viral titres)	HPP reduces CMV significantly, but not as effectively as HoP or HTST. More studies are needed to corroborate.

**Fig. 4:**
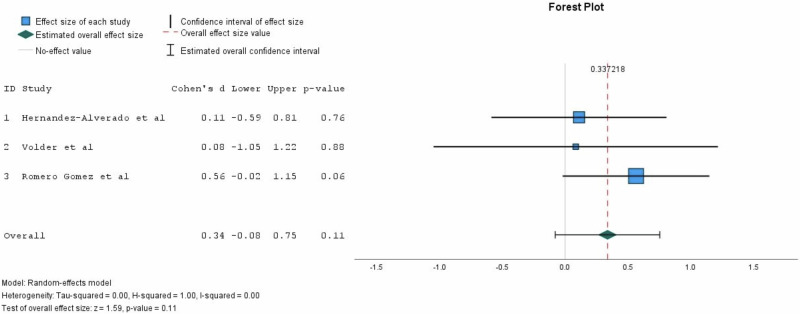
Meta-analysis: mean viral loads in breastmilk provided by transmitting mothers versus non-transmitting mothers. Mean viral loads and standard deviations from each study were used to perform a Cohen’s d meta-analysis with a random effects model. Pooled mean difference was not statistically significant (*P* = 0.11) and confidence intervals crossed the line no difference.

To quantify the relationship between viral loads in breastmilk and rates of transmission, a fixed effects meta-analysis was run on three articles reporting mean viral loads in breastmilk from transmitting vs non-transmitting mothers (Fig. 4). The difference in mean CMV viral load in viral loads breastmilk between transmitting and non-transmitting women was not significant (*P* = 0.11).

### Arms B and C: summary of findings

Sixteen articles were studied to assess the impact of breastmilk treatment methods on nutritional elements *viz*. lactose, lipids, and protein [11, 13, 44–57]. The results are summarised in Table 4. Freeze thawing at variable temperatures and durations do not have a significant effect on macronutrients of breastmilk. A single study reported an increase in free triglycerides [13]. Lactose remained most stable when milk samples were pasteurized. Six studies reported a statistically significant decrease in lipid content and 5 studies reported a small but statistically significant decrease in milk proteins. Novel processing methods of irradiation and HPP had no significant effect on macronutrients.

**Table 4:** Summary of effects of treatment methods on lipids, lactose and protein in breastmilk.

Treatment methods	Author	Effect of treatment on (% reduction)		
		Lactose	Lipids	Protein
Freeze-thawing	Friend et al. [43]	x	NS	x
(−20C for variable durations)	Yuen et al. [13]	NS	NS	NS
	Garcia-lara et al. [44]	NS	NS	NS
	Paduraru et al. [45]	NS	x	x
	Tanriverdi et al. [46]	NS	Significant decrease	NS
Freeze-Thawing (4C for variable durations)	Ezz El Dinn et al. [47]	NS	NS	NS
	Yuen et al. [13]	59% increase	Slight decrease	Slight decrease
	Paduraru et al. [45]	NS	NS	NS
Holder pasteurization	Garcia-lara et al. [44]	NS	0.062	x
	Meredith-Dennis et al. [48]	x	0.209	Significant decrease
	Lima et al. [49]	NS	NS	~2%
	Adhisivam et al. [51]	0.16	0.25	0.125
	Piemontese et al. [52]	0.0111	0.0549	0.0227
	Pitino et al. [53]	NS	NS	NS
	Lima et al. [50]	NS	NS	0.0126
	Chang et al. [11]	x	0.2045	NS
	Caballero Martin et al. [54]	NS	0.062	NS
	Lamb et al. [55]	NS	NS	NS
	Martysiak Zurowska et al. [56]	NS	NS	NS
High Temperature short time	Pitino et al. [53]	NS	NS	NS
Microwave irradiation	Martysiak Zurowska et al. [56]	NS	NS	NS
UV-C irradiation	Pitino et al. [53]	NS	NS	NS
High pressure processing	Pitino et al. [53]	Slight decrease	NS	NS

*NS* Not Statistically Significant, *x* Data not reported/available in article.

Nineteen published studies were reviewed to quantify impact of treatment methods on bioactive elements [12, 57–69]. The effect of each treatment method on different elements is summarized in Table 5. All articles studying the effect of HoP reported a significant decrease in lactoferrin, lysozyme, and sIgA content. Of these, 2 studies reported a near complete degradation of BSSL. HTST results in similar levels of degradation of these elements to HoP. Limited evidence for irradiation methods suggests a moderated decrease in lactoferrin, lysozymes and sIgAs. Three studies report conflicting results on the effect of HPP on bioactive elements. Compared to HoP, HPP largely preserves all concerned elements.

**Table 5:** Summary of effects of treatment methods on four bioactive elements of breastmilk.

Treatment methods	Author	Effect of treatment on: (% reduction)			
		Lactoferrin	Lysozyme	sIgA	BSSL
Freeze-thawing	Chang et al. [11]	0.115	0.398	0.082	x
	Akinbi et al. [58]	NS	0.32	0.51	x
	Berkow et al. [59]	NS	x	x	No effect
Holder pasteurisation	Klotz et al. [12]	0.68	0.28	0.17	0.996
	Martysiak Zurowaska et al. [56]	0.6	0.44	0.58	x
	Daniels et al. [60]	0.289	Statistically Significant Decrease	0.211	x
	Chang et al. [11]	0.66	Statistically Significant Decrease	0.259	x
	Zhang et al. [61]	0.8	0.648	0.515	0.9858
	Akinbi et al. [58]	0.44	0.6	0.6	x
	Aceti et al. [62]	0.875	x	x	x
	Baro et al. [64]	x	x	x	1
	Paulaviciene et al. [65]	0.8	0.35	0.05	0.992
	Daniels et al. [60]	0.614	0.252	0.748	x
High temperature short time	Aceti et al. [62]	0.835	x	Statistically significant Decrease	x
	Chantry et al. [63]	x	x	0.2	x
	Baro et al. [64]	x	x	x	Activity present
	Goldblum et al. [66]	x	x	x	0.97
UV-C irradiation	Christen et al. [67]	0.13	0.25	0.11	x
Microwave irradiation	Martysiak Zurowaska et al. [56]	0.5	0.3	0.09	x
High pressure processing	Zhang et al. [61]	0.48	Increase	0.022	No effect
	Aceti et al. [62]	Preserved	x	Statistically significant Decrease	x
	Viazis et al. [68]	x	4.2% reduction after 120 min	Statistically Significant Decrease	x

*NS* Not Statistically Significant, *x* Data not reported/available in article.

## Discussion

To our knowledge, this is the first study to combine, quantify and review the impact of breastmilk treatment methods on pCMV rates, viral burden and nutritive and bioactive elements. Additionally, High Pressure Processing (HPP), a treatment method commonly used in the food processing industry but very recently considered for CMV eradication [10] has been included.

In our systematic review of 26 articles, a cumulative number of 3024 preterm infants and breastmilk from 251 mothers were included to assess pCMV infection, transmission, and viral loads.

Pasteurisation methods, including HoP and other variations reduced rates of pCMV in vulnerable infants by 82% compared to untreated (fresh) milk, consistent with Lanzieri et al. [1]. This concurs with in-vitro experiments demonstrating complete eradication of CMV from breastmilk undergoing HoP [33] which impacts the transmission of CMV via breastmilk. Freeze thawing reduced pCMV infection rate by 53% compared to untreated breastmilk, representing a less effective method of reducing CMV transmission to the vulnerable infant.

We estimated the percentage change in the amount of CMV in breastmilk before and after treatment via various methods. HTST, HPP and microwave irradiation (750 W, 30 s) reduce CMV infectivity by about 99 to 100%. While UV-C reduces CMV in breastmilk by 54.6%, it is less effective than HoP. Similar results were highlighted by Bardanzellu et al. in their narrative review on pCMV [70]. However, there are no clinical studies on CMV elimination via non-thermal methods.

Clinical studies on pCMV transmission reported a statistically significant difference between the magnitude of viral loads in breastmilk of successfully transmitting mothers compared to non-transmitting counterparts [28, 34, 35, 39]. This would imply the existence of a viral load threshold, below which the risk of transmission of pCMV is low, and therefore safer for the vulnerable infant. To explore this hypothesis, a comparative meta-analysis was performed, which showed that the difference was not significant. Thus, attempts to reduce maternal CMV viral load in breastmilk may not be sufficient to reduce risk of CMV acquisition.

We report that macronutrient content of breastmilk remains stable under treatment by freeze thawing, irradiation (microwave and UV-C) and HPP. Most studies report no significant changes in lactose, lipid or protein content of breastmilk that is responsible for growth of the vulnerable infant. HoP appears to have the most significant impact on reducing some studied nutritional indices in breastmilk. Protein and lipid content is affected more than lactose. Although Wesolowska et al. did not include freeze-thawing in their study, they stated that carbohydrates are reduced by HTST and HoP [10]. This difference could be attributed to difference in milk processing times, and difference in sample preparation protocols.

Lactoferrin, lysozyme and sIgAs in breastmilk are most affected by HoP and HTST, followed by microwave and UV-C irradiation, consistent with findings of Wesolowska et al. [71]. This is attributed to heat sensitive denaturation of their protein structure on exposure to temperatures of 62.5 °C or higher [50]. However, some studies state that lysozyme is heat stable at controlled pH environments [69]. The possibility of pH variations confounding the effect of a treatment method cannot be excluded with current literature.

Although clear evidence supporting the following is limited, irradiation methods (microwave, UV-C) better preserve bioactive elements compared to HoP [68]. HPP has relatively negligible effects on lysozymes, sIgAs and BSSL, which were denatured by all heating and irradiation methods. This is also validated by Wesolowska et al. [71]. However, while included studies reported concentration of these elements in their samples, whether their functionality was retained post-treatment is unknown.

In the Neonatal Intensive Care Unit (NICU), a preterm or VLBW infant is routinely provided with parenteral nutrition until they are capable of milk feeding. Milk fortification also serves to artificially increase the energy content of administered feeds [72]. If so, it is plausible that for a defined period (2–6 weeks), the reduced nutritional value of ‘treated’ breast milk, if administered, may not have a significant effect on the overall growth of the premature baby, but may adequately minimise the risk of CMV exposure. A consideration may therefore be to provide ‘treated’ breast milk for the period of highest CMV virus excretion (virolactia) deemed as the first 2–6 weeks post-birth [41]. Clinicians treating babies most at risk of pCMV may strategize by choosing to prioritize CMV elimination with preservation of bioactive elements over nutrition for this higher risk period, (first 2–6 weeks post-birth) for the high risk, most premature infants.

Necrotising enterocolitis is a common complication of prematurity that has a high fatality and morbidity rate. Provision of human milk to the at-risk infant has a protective dose-response effect due to the presence of sIgAs, lactoferrin, lysozymes, BSSL and other factors. Intestinal microbiota, shaped by breastmilk, also play a role in preventing NEC [72]. Therefore, choosing to treat breastmilk to reduce the risk of any infection, including pCMV, should ideally place emphasis on preserving bioactive elements. Microwave irradiation at high powers and HTST can be viable treatment methods in this regard.

In resource limited settings, pasteurization (HoP and HTST) may be the only viable method of breastmilk treatment. While these methods can reliably eradicate CMV, the treating clinician should consider the loss of nutritional and bioactive value of the treated breastmilk and supplement as required. Although microwave irradiation can be a potential alternative, more in-vitro studies are required to verify its efficacy in eliminating CMV from breast milk.

High Pressure Processing has gained traction over recent years as a potential alternative to HoP that eradicates breastmilk CMV as well as preserves nutritional and bioactive elements [2, 9]. Sealed packets of breastmilk are placed in a vessel with compression fluid, designed to create a closed system. Increased pressures via HPP induced disruption of gene expression, protein synthesis and key metabolic reactions [73]. However, the infrastructure required for a hydraulic pressure plant poses a significant challenge for hospitals to implement it as a routine procedure. This method may, however, be a promising alternative at human milk banks that currently process large amounts of breastmilk using HoP.

### Limitations

There have been no randomised clinical trials for inclusion in this study. All extracted articles were cohort and observational studies, with varied levels of risk of bias. Additionally, some conversion factors used in this study are mathematical estimates, which do not consider differences in assay protocols or sample preparation methods that can occur with a laboratory experiment. Randomized controlled trials focused on measuring quantitative impact of a treatment on breastmilk components and rate of transmission will be instrumental to corroborate the results of this study.

A major limitation that underlies any analyses regarding breastmilk is the possible combination of treatment methods and variations during the intervention. In clinical studies observing pCMV incidence, milk from seropositive mothers was frozen at (−20 °C) or lower for several days [4, 74]. To perform laboratory experiments, donor milk samples were frozen for up to 48 h and transported before usage. Once irradiated or pasteurised, methods employed to cool the milk samples can also affect the viability of milk components or CMV. With current study designs, isolated effect of each treatment method cannot be explored.

## Conclusion

This study is a comprehensive, multifactorial analysis of the current literature on the impact of breastmilk treatment methods on viral, nutritive, and immunological elements. HoP is the gold standard treatment method to reduce CMV infectivity. HoP eliminates all CMV in milk samples. High Temperature Short Time, High Pressure Processing, and microwaves display high efficacy in reducing CMV viral loads in breastmilk. While macronutrients are relatively stable under all treatment methods, bioactive elements undergo significant degradation by HoP and HTST. With more studies to corroborate results, microwave irradiation and High-Pressure Processing hold promise as alternative treatment methods that eliminate CMV in breastmilk, while preserving bioactive elements necessary for the growth and development of the vulnerable infant. Strategies that limit the period of treatment of breast milk just for the period of high CMV breast milk excretion (first 2 to 4 or 6-weeks post-partum) may represent a potential strategy for consideration.

## Supplementary information

Supplementary Material
