# Supplementary material for: Strategies to reduce CMV infectivity in breastmilk to preterm babies – impact on transmission, nutrients, and bioactivity: a systematic review and meta-analysis
Source: J Perinatol. 2025 Mar 27;45(4):426–37. doi: 10.1038/s41372-025-02254-9 (PMC12069081; doi:10.1038/s41372-025-02254-9)
Supplement: Supplementary file 1 — Supplementary Material [file 41372_2025_2254_MOESM1_ESM.docx]

## Supplementary Material 1: Search Strategies for Systematic Review – Arm 1

*EMBASE: 1974 to 2022 August 31*

| **#** | **Query** | **Results from 1 Jun 2022** |
| --- | --- | --- |
| 1 | exp Food Preservation/ or Food Handling/ or Milk Hygiene/ | 31,437 |
| 2 | exp Freeze thawing/ or Irradiation/ or exp Microwave Irradiation/ or exp Ultraviolet irradiation/ | 88,359 |
| 3 | (Freez$ or Freeze-thaw$ or Frozen).tw. | 158,121 |
| 4 | (Pasteuriz$ or Pasteuris$ or heating or heat treatment or thermal processing or thermal treatment).tw. | 75,750 |
| 5 | (Low Temperature Long Time or Holder Pasteurization or Holder Treatment).tw. | 187 |
| 6 | (HTST or short term pasteurization or short term pasteurisation or Ultrashort treatment or Flash Heating).tw. | 338 |
| 7 | (UV-C or UV-C irradiation).tw. | 1,833 |
| 8 | (Irradiation or Ultraviolet).tw. | 242,666 |
| 9 | (Microwav$ or Microwave treatment or Microwave Irradiation).tw. | 40,920 |
| 10 | (Irradiat$ or non-thermal treatment or non thermal treatment).tw. | 237,107 |
| 11 | (Cold Pasteurization or Cold Pasteurisation).tw. | 24 |
| 12 | (HPP or High pressure treatment or High Pressure or Hydrostatic Pressure Treatment).tw. | 28,775 |
| 13 | 1 or 2 or 3 or 4 or 5 or 6 or 7 or 8 or 9 or 10 or 11 or 12 | 604,768 |
| 14 | Milk/ or Cow Milk/ or exp Milk by preservation/ | 60,410 |
| 15 | exp Boiled milk/ or exp Pasteurised Milk/ or exp UHT milk/ | 304 |
| 16 | Milk Bank/ | 366 |
| 17 | Breast Milk/ or Donor Milk/ | 25,258 |
| 18 | Breast Feeding/ or Breast Milk Expression/ | 51,156 |
| 19 | exp Breastfeeding transmission/ | 61 |
| 20 | Milk donor/ | 93 |
| 21 | (Breastmilk or Breast Milk or Breastfeeding or donor milk or treated milk or milk banks or breastmilk transmission or Breast milk transmission or vertical transmission).tw. | 56,082 |
| 22 | 14 or 15 or 16 or 17 or 18 or 19 or 20 or 21 | 142,290 |
| 23 | 13 and 22 | 6,901 |
| 24 | exp Cytomegalovirus/ or exp Human Cytomegalovirus/ or exp Cytomegalovirus infection/ | 62,396 |
| 25 | (Cytomegalovirus or Cytomegalovirus infection$ or CMV or HCMV).tw. | 65,648 |
| 26 | (Cytomegalovirus or Cytomegalovirus infection$ or CMV or HCMV or CMV infection$).tw. | 65,648 |
| 27 | (pCMV or Postnatal CMV or Postnatal Cytomegalovirus).tw. | 1,570 |
| 28 | (Post-natal CMV or Post natal CMV or Post-natal cytomegalovirus or Post natal cytomegalovirus).tw. | 10 |
| 29 | ((Postnatal$ or Post-natal$ or Post natal$) adj4 (CMV or Cytomegalovirus or Cytomegalovirus Infection$ or HCMV)).tw. | 267 |
| 30 | ((Postnatal acquisition or Post-natal acquisition) adj2 (cytomegalovirus or CMV or HCMV or cytomegalovirus infection)).tw. | 7 |
| 31 | ((Breastmilk acqui$ or breast milk acqui$) adj2 (Cytomegalovirus or Cytomegalovirus infection or CMV or HCMV)).tw. | 23 |
| 32 | cytomegalovirus transmission.tw. | 67 |
| 33 | 24 or 25 or 26 or 27 or 28 or 29 or 30 or 31 or 32 | 85,140 |
| 34 | 23 and 33 | 119 |

*MEDLINE® All including Epub Ahead of Print,In Process & Other Non-Indexed Citations, Daily and Versions ® 1946-current*

| **#** | **Query** | **Results from 31 May 2022** |
| --- | --- | --- |
| 1 | exp Food Handling/ | 48,398 |
| 2 | exp Pasteurization/ | 2,104 |
| 3 | exp Food Preservation/ | 10,238 |
| 4 | exp Food Irradiation/ | 1,089 |
| 5 | exp Freezing/ | 11,019 |
| 6 | exp Cryopreservation/ or exp Freeze drying/ | 30,692 |
| 7 | Preserv$.tw. | 223,156 |
| 8 | (Freez$ or Freeze-thaw$ or Frozen).tw. | 89,062 |
| 9 | (Pasteuriz$ or Pasteuris$ or heating or heat treatment or thermal processing or thermal treatment).tw. | 44,917 |
| 10 | (Low Temperature Long Time or Holder Pasteurization or Holder Treatment).tw. | 152 |
| 11 | (HTST or short term pasteurization or short term pasteurisation or Ultrashort treatment or Flash heating).tw. | 247 |
| 12 | (UV-C or UV-C irradiation).tw. | 1,394 |
| 13 | (Irradiation or Ultraviolet).tw. | 144,865 |
| 14 | (Microwave$ or Microwave treatment or Microwave irradiation).tw. | 20,497 |
| 15 | (Irradiat$ or non-thermal treatment or non-thermal or non thermal treatment).tw. | 138,916 |
| 16 | (Cold Pasteurization or Cold Pasteurisation).tw. | 20 |
| 17 | (HPP or High Pressure treatment or High Pressure or Hydrostatic pressure treatment).tw. | 16,476 |
| 18 | 1 or 2 or 3 or 4 or 5 or 6 or 7 or 8 or 9 or 10 or 11 or 12 or 13 or 14 or 15 or 16 or 17 | 596,533 |
| 19 | exp Milk/ or exp Colostrum/ or exp Mothers/ | 106,004 |
| 20 | exp Milk, Human/ | 13,135 |
| 21 | exp Milk Proteins/ | 25,284 |
| 22 | exp Breast Feeding/ | 28,488 |
| 23 | exp Breast Milk Expression/ | 359 |
| 24 | exp Milk Banks/ | 595 |
| 25 | (Breastmilk or Breast Milk or Breastfeeding or Breast Feeding or donor Milk or raw milk or Untreated (fresh) milk or treated milk or milk banks or breast milk transmission or Vertical Transmission).tw. | 45,093 |
| 26 | exp Infectious Disease Transmission, Vertical/ | 17,470 |
| 27 | 19 or 20 or 21 or 22 or 23 or 24 or 25 or 26 | 163,352 |
| 28 | 18 and 27 | 9,879 |
| 29 | exp Cytomegalovirus/ | 15,571 |
| 30 | exp Cytomegalovirus Infections/ | 18,090 |
| 31 | (Cytomegalovirus or Cytomegalovirus Infection$ or CMV or HCMV).tw. | 37,325 |
| 32 | (Cytomegalovirus or Cytomegalovirus Infection$ or CMV or HCMV or CMV infection).tw. | 37,325 |
| 33 | (pCMV or postnatal CMV or Postnatal Cytomegalovirus).tw. | 1,023 |
| 34 | (Post-natal CMV or Post natal CMV or Post-natal cytomegalovirus or Post natal cytomegalovirus).tw. | 6 |
| 35 | ((Postnatal$ or Post-natal$ or Post natal$) adj4 (CMV or Cytomegalovirus or Cytomegalovirus infection$ or HCMV)).tw. | 176 |
| 36 | ((Postnatal acquisition or post-natal acquisition) adj2 (cytomegalovirus or CMV or HCMV or cytomegalovirus infection)).tw. | 5 |
| 37 | ((Breastmilk acqui$ or breast milk acqui$) adj2 (Cytomegalovirus or Cytomegalovirus infection or CMV or HCMV)).tw. | 17 |
| 38 | Cytomegalovirus transmission.tw. | 56 |
| 39 | 29 or 30 or 31 or 32 or 33 or 34 or 35 or 36 or 37 or 38 | 40,551 |
| 40 | 28 and 39 | 75 |

## Supplementary Material 2: Individual Proportional Meta-analysis plots.

*
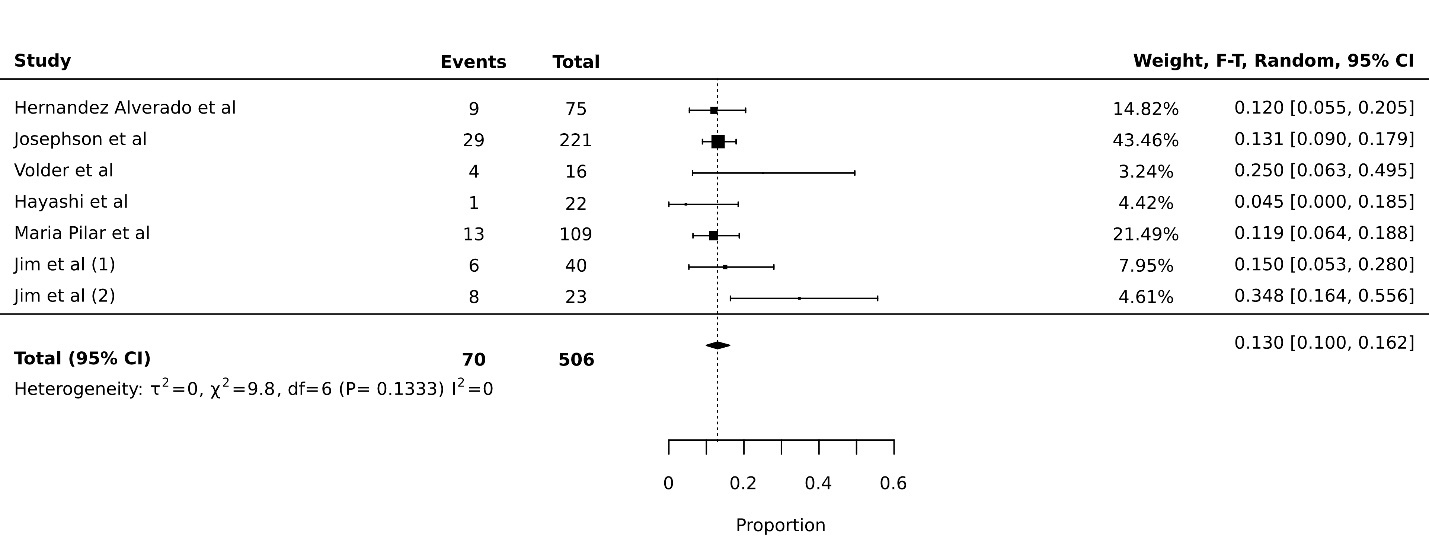
*

*Figure 5: Pooled Proportions of postnatal cytomegalovirus infections in infants fed Untreated (fresh) milk. The pooled number of infected infants is 70, and net proportion of transmission is 13%.*

*
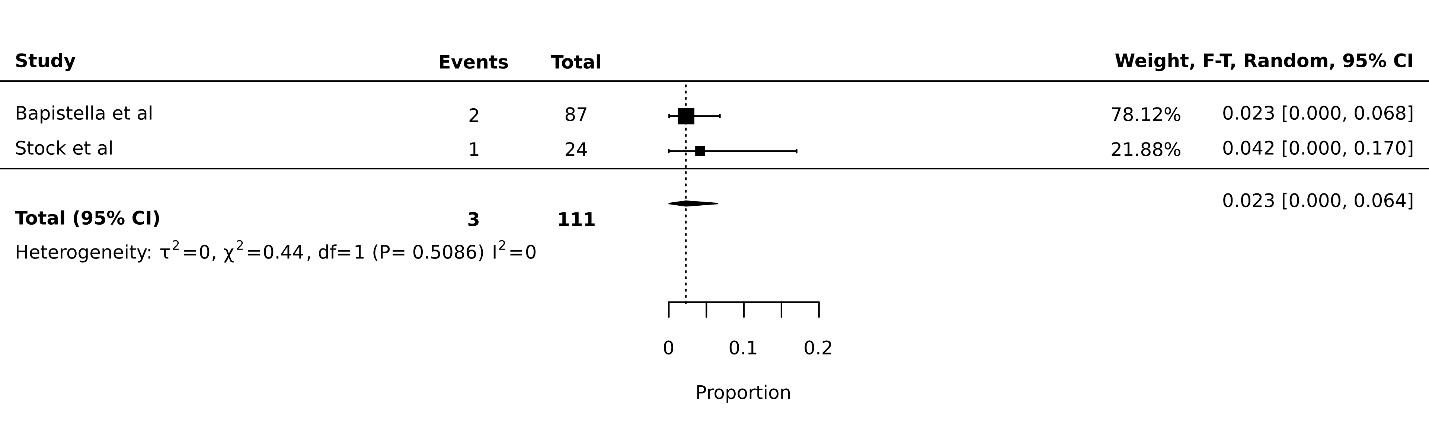

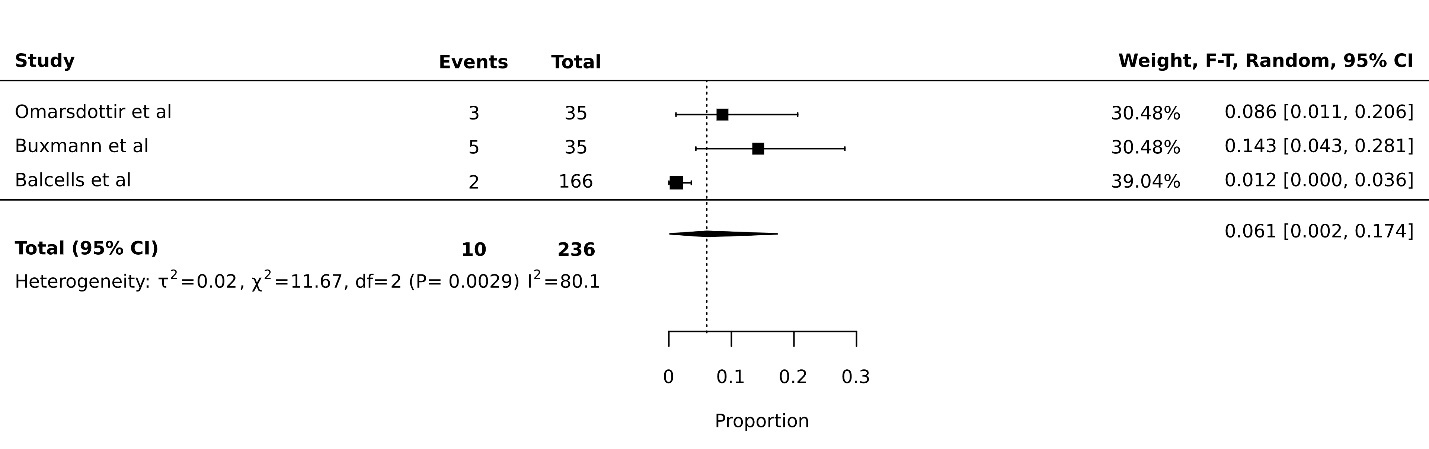
Figure 6: Pooled Proportions of postnatal Cytomegalovirus infections in infants fed frozen milk. The pooled number of infected infants is 10, and net proportion of transmission is 6.1%*

*Figure 7: Pooled Proportions of postnatal Cytomegalovirus infections in infants fed pasteurised milk. The pooled number of infected infants is 3, and net proportion of transmission is 2.3%*

## Supplementary Material 3a: Data tables: Rates of transmission of CMV by treatment methods

| **S.No.** | **Author** | **Year** | **Treatment provided to milk samples** | **Total number of babies studied for the concerned outcome (n)** | **Infected Babies (x)** | **Proportion infected (f)= [(x/n)*100] (%)** |
| --- | --- | --- | --- | --- | --- | --- |
| 1 | Buxmann et al | 2009 | Freeze-thawing | 35 | 5 | 14.29 |
| 2 | Omarsdottir et al | 2015 | Freeze-thawing | 35 | 3 | 8.57 |
| 3 | Balcells et al | 2016 | Freeze-thawing | 166 | 2 | 1.2 |
|  |  |  |  |  |  |  |
| 4 | Yoo et al | 2015 | Pasteurization | 62 | 0 | 0 |
| 5 | Stock et al | 2015 | Pasteurization | 24 | 1 | 4.17 |
| 6 | Bapistella et al | 2019 | Pasteurization | 87 | 2 | 2.3 |
|  |  |  |  |  |  |  |
| 7 | Jim et al | 2004 | No treatment | 40 | 6 | 15 |
| 8 | Jim et al | 2009 | No treatment | 23 | 8 | 34.78 |
| 9 | Hayashi et al | 2011 | No treatment | 22 | 1 | Manually calculated: 4.55 |
| 10 | Josephson et al | 2014 | No treatment | 221 | 29 | 13.12 |
| 11 | Romero Gomez et al | 2015 | No treatment | 109 | 13 | 11.93 |
| 12 | Volder et al | 2021 | No treatment | 16 | 4 | 25 |
| 13 | Hernandez Alvarado et al | 2021 | No treatment | 75 | 9 | 12 |

*All manual calculations are detailed in Appendix F.*

## Supplementary Material 3b: Data Tables: Viral Infectivity of breastmilk by treatment method

| **S.No.** | **Author** | **Year** | **Treatment provided to milk samples** | **Temperature/Duration conditions** | **Initial Viral Load (copies/mL or PFU/mL)** | **Final Viral Load (copies/mL or PFU/mL)** | **Mean Change of Viral infectivity (%)** |
| --- | --- | --- | --- | --- | --- | --- | --- |
| 1 | Welsh et al | 1979 | Pasteurization | 62.5°C, 30 minutes | NR | NR | Qualitative Statement: Complete elimination |
| 2 | Dworsky et al | 1982 | Freezing | (-20°C), upto 7 days | 1000 PFU/mL | NR | Qualitative Statement: CMV still present |
| 3 | Hamprecht et al | 2004 | Freezing | (-20°C), 4 days | 42200 copies/mL | 46633.33 copies/mL | 10.5% increase |
| 5 | Hosseini et al | 2016 | Freezing | (-20°C), 3 days | 76040 copies/mL | 6750 copies/mL | 91.1% reduction |
| 6 | Lloyd et al | 2016 | UV-C Irradiation | 254 nm, 10 seconds, at 1 cm and 5cm | 1540 PFU/mL | 490 PFU/mL | 68.2% reduction |
| 7 | Shoshan et al | 216 | Microwave Irradiation | 500 W and 700 W, 30 seconds. | NR | NR | Qualitative Statement: Significant CMV reduction |
| 8 | Maschmann et al | 2019 | HTST | 72°C, 5 seconds | NR | NR | 100% reduction |
| 9 | Mikawa et al | 2019 | Microwave Irradiation | 500 W, 20, 30, 40 and 60 seconds | 772 PFU/mL | 257 PFU/mL (at 20s), 0 (at 30s) | 66.7% reduction at 20s, 100% after 30s |
| 10 | Gaya et al | 2021 | Holder Pasteurization | 63°C, 30 minutes | 500,000 PFU/mL | 0 | 100% reduction |
| 11 | Pitino et al | 2022 | HPP | 330MPa, 500MPa, 600MPa for 8 and 10 mins each | 12,5892.54 PFU/mL | 4 PFU/mL | 99.99% reduction in all conditions |
| 12 | Sam et al | 2018 | Freezing | (-20°C) uptil Day 90 | 9,236.93 copies/mL | 6847.44 copies/mL | 25.8% reduction |
| ***Legend:***  ***W – Watts (Unit of power)***  ***PFU/mL – Plaque Forming Units/mL***  ***NR – Not Reported***  ***Green tabs – information in the tabs have been calculated manually. For further calculations see Appendix F.*** | | | | | | | |

## Supplementary Material 3c: Mean Viral Loads of CMV in breastmilk by transmission status.

| **S.No.** | **Author** | **Year** | **Mean Viral Load in breastmilk of transmitting mothers (copies/mL)** | **Standard Deviation** | **Mean Viral Load in breastmilk of non-transmitting mothers (copies/mL)** | **Standard Deviation** |
| --- | --- | --- | --- | --- | --- | --- |
| 1 | Hernandez Alverado et al | 2021 | 54,495 | 60,627 | 41,396 | 124,182 |
| 2 | Volder et al | 2021 | 65,675 | 25,546.94 | 25,366 | 44,831.83 |
| 3 | Romero Gomez et al | 2011 | 2.6 * 10^5 | 7.6 * 10^5 | 5.7 * 10^4 | 2.7 * 10^5 |
| ***Legend:***  ***Green tabs show manually calculated data points. Further calculations are detailed in Appendix F.*** | | | | | | |

## Supplementary Material 4: Manual Calculations for all highlighted measurements.

**Calculations for Appendix C: Data Tables**

1. Jim et al (2004)

Given number of infants infected = 4
Given number of infants exposed to CMV = 40

Proportion of CMV infected infants = $\frac{6}{40}\times100=0.15\times100=15\%$

1. Jim et al (2009)

Given number of infants infected = 8
Given number of infants exposed to CMV = 23

Proportion of CMV infected infants = $\frac{8}{23}\times100=0.347\times100=34.8\%\approx35\%$

**Calculations for Appendix D: Data Tables**

1. Dworsky et al

Given initial viral load = 200 PFU/0.2 mL
To convert to PFU/mL:

$$\frac{200 PFU}{0.2 mL}=\frac{200\times10}{2}\left( \frac{PFU}{mL} \right)=1000\frac{PFU}{mL}$$

1. Hamprecht et al (2005)

To calculate mean initial viral load:

Given viral load of milk sample 1 = 56300 copies/mL
Given viral load of milk sample 2 = 49000 copies/mL
Given viral load of milk sample 3 = 21300 copies/mL

$$Mean Viral Load=\frac{56300+49000+21300}{3} \left( \frac{copies}{mL} \right)= 2200 \left( \frac{copies}{mL} \right)$$

To calculate final viral load:

Given viral load of treated milk sample 1 = 62100 copies/mL
Given viral load of treated milk sample 2 = 51900 copies/mL
Given viral load of treated milk sample 3 = 25900 copies/mL

$$Mean Viral Load=\frac{62100+51900+25900}{3} \left( \frac{copies}{mL} \right)= 4633.33 \left( \frac{copies}{mL} \right)$$

$$Change in viral infectivity=\frac{final-initial}{initial}\times100$$

$$Change in viral infectivity=\frac{4633.33-2200}{4633.33}\times100=10.5\% increase$$

1. **Hosseini et al**

Given initial mean viral load = 76.04 copies/uL

To convert to copies/mL:

$$\frac{76.04 copies}{\mu L}=76.04\times1000\left( \frac{copies}{mL} \right)=76040 \frac{copies}{mL}$$

Given final mean viral load = 6.75 copies/uL

To convert to copies/mL:

$$\frac{6.75 copies}{\mu L}=6.75\times1000\left( \frac{copies}{mL} \right)=6750 \frac{copies}{mL}$$

$$Change in viral infectivity=\frac{final-initial}{initial}\times100$$

$$Change in viral infectivity=\frac{76040-6750}{76040}\times100=91.12\% decrease$$

1. **Lloyd et al**

Given initial spiked viral load = 2200 TCID_50_/mL

We know, $\frac{1PFU}{ml}=0.7 \times\frac{1TCID50}{mL}$

$$Initial Viral Load=0.7\times2200\frac{TCID50}{ml}=1540\frac{PFU}{mL}$$

Similarly, given final viral load = 700 CID_50_/mL at 5cm from source.

$$Final Viral Load=0.7\times700\frac{TCID50}{ml}=490\frac{PFU}{mL}$$

$$Change in viral infectivity=\frac{final-initial}{initial}\times100$$

$$Change in viral infectivity=\frac{490-1540}{490}\times100=68.2\% decrease$$

1. **Mikawa et al**

Given initial mean viral load = 772 PFU/mL
Given final mean viral load = 257 PFU/mL at 20 seconds of treatment

$$Change in viral infectivity=\frac{final-initial}{initial}\times100$$

$$Change in viral infectivity=\frac{257-772}{772}\times100=66.7\% decrease$$

1. **Sam et al**

Given mean viral load at Day 0 (initial, untreated) = 3.73 log_10_ IU/mL

$$Mean viral load={10}^{3.73}=5370.31\frac{IU}{mL}$$

But we know,

$$1\frac{IU}{mL}=1.72 CMV\frac{copies}{mL}$$

$$Mean viral load=5370.31\times1.72=9236.93\frac{copies}{mL}$$

Given mean viral load on Day 4 (final, treated) = 3.60 Log_10_ IU/mL

$$Mean viral load={10}^{3.60}=3981.07\frac{IU}{mL}$$

Again,

$$1\frac{IU}{mL}=1.72 CMV\frac{copies}{mL}$$

$$Mean viral load=3981.07\times1.72=6847.44\frac{copies}{mL}$$

$$Change in viral infectivity=\frac{final-initial}{initial}\times100$$

$$Change in viral infectivity=\frac{6847.44-9236.93}{9236.93}\times100=25.8\% decrease$$

1. **Pitino et al**

Given initial viral load = 5.1 log_10_ PFU/mL

$$Mean viral load={10}^{5.1}=125892.54\frac{PFU}{mL}$$

Given final viral load = 4 PFU/mL

$$Change in viral infectivity=\frac{final-initial}{initial}\times100$$

$$Change in viral infectivity=\frac{4-125892.54}{125892.54}\times100=99.996\% decrease$$

**Calculations for Appendix E: Data Tables**

1. **Volder et al**

Given,
Sample size of non-transmitting women’s milk samples = 4
Mean viral load in samples = 65675 copies/mL
95% Confidence Intervals = (40,639 – 90711)

We know,

$$Standard Deviation \left( SD \right)= \sqrt{N}\times\left[ \frac{Upper Limit-Lower Limit}{3.92} \right]$$

$$SD=\sqrt{4}\times\left[ \frac{90711-40639}{3.92} \right]=2\times12773.46=25546.94$$

Given,
Sample size of non-transmitting women’s milk samples = 12
Mean viral load in samples = 25366 copies/mL
95% Confidence Intervals = (5102 – 55834)

We know,

$$Standard Deviation \left( SD \right)= \sqrt{N}\times\left[ \frac{Upper Limit-Lower Limit}{3.92} \right]$$

$$SD=\sqrt{12}\times\left[ \frac{55834-5102}{3.92} \right]=3.46\times12941.83=44831.83$$
